# Supplementary material for: Membranous Expression of Heart Development Protein with EGF-like Domain 1 Is Associated with a Good Prognosis in Patients with Bladder Cancer
Source: Diagnostics (Basel). 2023 Sep 27;13(19):3067. doi: 10.3390/diagnostics13193067 (PMC10572329; doi:10.3390/diagnostics13193067)
Supplement: Supplementary file 1 [file diagnostics-13-03067-s001.zip › diagnostics-2599718-supplementary.pdf]

**Supplementary Figure**

Supplementary Figure S1

Figure S1. Probability of survival in patients with urothelial carcinoma of the bladder according to cytoplasmic HEG1 expression estimated using the Kaplan–Meier method. (A) Cancer-specific survival, (B) Recurrence-free survival

(A)

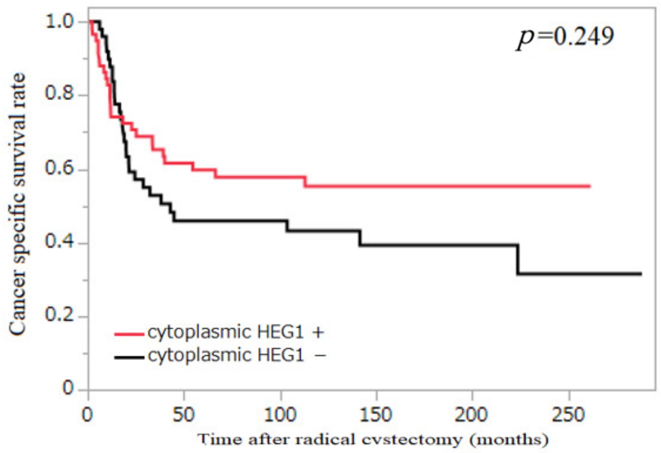


| Number at risk | | | | | | |
|---|---|---|---|---|---|---|
| cytoplasmic HEG1 + | 59 | 33 | 24 | 13 | 5 | 1 |
| cytoplasmic HEG1 - | 51 | 20 | 17 | 10 | 6 | 1 |

(B)

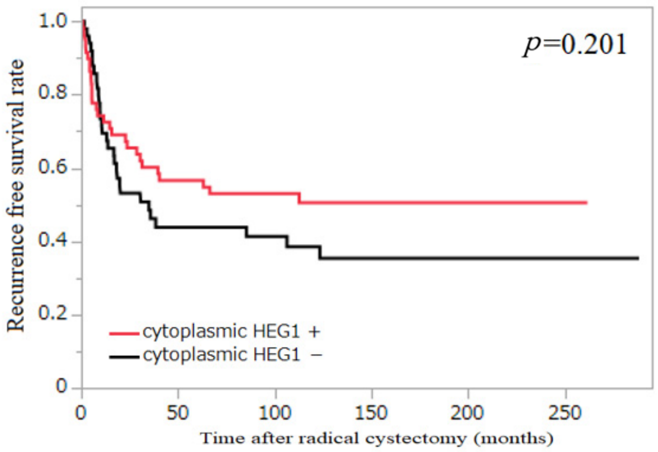


| Number at risk | | | | | | |
|---|---|---|---|---|---|---|
| cytoplasmic HEG1 + | 59 | 31 | 24 | 13 | 5 | 1 |
| cytoplasmic HEG1 - | 51 | 19 | 15 | 8 | 5 | 1 |
